# Recent Advances in the Endogenous Brain Renin-Angiotensin System and Drugs Acting on It

**DOI:** 10.1155/2021/9293553

**Published:** 2021-11-30

**Authors:** Aswar Urmila, Patil Rashmi, Ghag Nilam, Bodhankar Subhash

**Affiliations:** Department of Pharmacology, Poona College of Pharmacy, Bharati Vidyapeeth (Deemed to be University), Erandwane, Pune 411038, India

## Abstract

The RAS (renin-angiotensin system) is the part of the endocrine system that plays a prime role in the control of essential hypertension. Since the discovery of brain RAS in the seventies, continuous efforts have been put by the scientific committee to explore it more. The brain has shown the presence of various components of brain RAS such as angiotensinogen (AGT), converting enzymes, angiotensin (Ang), and specific receptors (ATR). AGT acts as the precursor molecule for Ang peptides—I, II, III, and IV—while the enzymes such as prorenin, ACE, and aminopeptidases A and N synthesize it. AT1, AT2, AT4, and mitochondrial assembly receptor (MasR) are found to be plentiful in the brain. The brain RAS system exhibits pleiotropic properties such as neuroprotection and cognition along with regulation of blood pressure, CVS homeostasis, thirst and salt appetite, stress, depression, alcohol addiction, and pain modulation. The molecules acting through RAS predominantly ARBs and ACEI are found to be effective in various ongoing and completed clinical trials related to cognition, memory, Alzheimer's disease (AD), and pain. The review summarizes the recent advances in the brain RAS system highlighting its significance in pathophysiology and treatment of the central nervous system-related disorders.

## 1. Introduction

The renin-angiotensin system (RAS) is of paramount importance, having a role in the regulatory pathway involved in the maintenance of blood pressure (BP), body fluid volume, and sodium homeostasis. Conventional RAS involves the conversion of inactive angiotensinogen into angiotensin I (Ang I) in the presence of renin which is released from the kidney in response to low blood volume. Angiotensin-converting enzyme (ACE) converts Ang I into angiotensin II (Ang II) which acts on an angiotensin type 1 (AT1) and angiotensin type 2 (AT2) receptor. AT1 and AT2 are involved in various physiological changes such as an increase in BP, volume overload, and facilitation of aldosterone release. Intrinsic brain RAS is an enzyme-neuropeptide system having functional components (angiotensinogen, peptidases, angiotensin, and specific receptor proteins) with important biological and neurobiological activities in the brain.

## 2. History of Brain RAS

Renin was first named as a kidney hormone by Tigerstedt and Bergman in the year 1898, where they observed its pressure effects in rabbits [1]. Angiotensin was discovered during the late 1930s concurrently by Page in the United States and by Braun-Menenez and his colleagues in South America [2]. Ganten et al. in 1970 proposed that even after nephrectomy of adult mongrel dogs, the tissue's renin activity persisted even after 12 days [3]. As plasma renin is unable to cross the blood-brain barrier, it was predicted that there is the existence of brain RAS independent of the kidney.

Philips and Felix [4] proved the presence of Ang II-activated neurons in the brain and subfornical organs [4]. The results indicated that the components of the renin/angiotensin system are available at the level of the brain cell itself. All the components of brain RAS such as enzyme isorenin, ANG, Ang I, converting enzymes, and Ang II are found in the brain [5–9]. Brain RAS and circulating RAS have been proven to be independent of each other [10–12]. Glial cells are identified as the site of synthesis of AGT [13]. These RAS peptides are present in astrocytes, glial cells, oligodendrocytes, and neurons of various areas of the brain [14, 15] such as the basal ganglia, cerebral cortex, and hippocampus [16]. The review explains the recent advances in the brain RAS system. It also highlights the status of the agonist and antagonist of brain RAS in the treatment of various neurological disorders.

### 2.1. Angiotensinogen

AGT is a high molecular weight molecule (49.548), made up of 453 amino acids (aa) synthesized in the liver. It is similar to *α*1 antitrypsin and is affiliated to the Serpin gene family [17]. It is present centrally in the brain with about 90% being expressed in astroglial cells and in some neurons present in regions of the brain controlling cardiovascular and other metabolic functions [18, 19]. However, renin is endogenously expressed in the brain, and increased brain RAS activity is also reported for inducing moderate hypertension; certain experimental evidence also suggests that AGT expression in the brain and circulating AGT regulation is an independent phenomenon [20]. For example, ACE blockade for a long time causes the lowering of AGT levels in plasma, but not in cerebrospinal fluid (CSF). It is suggested that Ang I is not formed in the brain, and availability of Ang II might be because of its excess from periphery to the brain, as it itself is BBB destructive [21]. However, it should be noted that some studies have identified AGT- and Ang II-positive cells in the brain [22]. Ang II exhibits a vasoconstrictor effect when acting on the AT1 receptor and vasodilator effect through the AT2 receptor [23] in the brain.

### 2.2. Ang III and Ang IV

Ang II in the presence of aminopeptidase A (APA) gets converted into Ang III, which is abundantly found in the brain [24]. Ang III is further converted into Ang IV by aminopeptidase N (APN) and aminopeptidase B (APB). Ang III acts through the AT2 receptor and has a main role in the regulation of BP and homeostasis [25]. Ang IV has the ability to bind to the AT1 and AT4 receptors. Ang IV when bound to the AT1 receptor showed the vasoconstrictor effect, and with the AT4 receptor, it improved learning and memory response [26]. Cognition enhancement is mainly because of its ability to increase cholinergic neurotransmission in the hippocampal region [27]. APA inhibitors, amastatin and RB150 (orally active), inhibit the conversion of Ang II to III in the brain thereby increasing Ang II which decreases BP and reduces the pressor response [28] via the AT2 receptor. The preclinical safety and efficacy of RB150 have taken it to a phase I study, where it (RB150/QGC001) was found to be clinically and biologically well-tolerated in healthy volunteers after single oral administrations up to 2000 mg. Thus, APA inhibitors open a new avenue as an oral antihypertensive drug segment.

In contrast, when APN (inhibiting metabolism of Ang III) was blocked by intracerebroventricular (Icv) injection of PC18 (2-amino-4-methylsulfonyl butane thiol), this resulted in rise in BP due to a central mechanism of Ang III. This effect was blocked by the AT1 antagonist losartan suggesting involvement of the AT1 receptor in producing the pressor response by Ang III [29].

### 2.3. Converting Enzymes of the Brain RAS: Renin/Prorenin

The active form of renin in the brain is prorenin. It is present in a low concentration called as prorenin. In the brain, it is present within neurons and astrocytes in two forms: intracellular renin and secreted renin (prorenin) [30, 31]. Prorenin acts on a prorenin receptor, activation of which leads to the generation of angiotensin peptides which activate the AT1 receptor resulting in GPCR signaling [32].

### 2.4. ACE and ACE2

Brain ACE is a key component of the brain RAS and is expressed in many regions of the brain (endothelium of central vasculature, choroid plexus, and astrocytes) especially in the regulation of BP [32]. It is a dipeptidyl peptidase in nature and hydrolyzes bradykinin, tachykinin, or substance P in the brain [33]. ACE cleaves Ang I to the active octapeptide Ang II. Icv infusion of ACE into the brain of Sprague-Dawley rats has shown to increase Ang II levels in the CSF leading to the increase in BP [32]. The ACE inhibitor captopril when administered centrally normalizes BP and renal sympathetic nerve activity hence improving brain RAS function [34]. ACE2, a form of ACE, was first identified in the year 2000 [35, 36]. The human ACE2 (hACE2) protein is a zinc metallopeptidase with 805 amino acids. ACE2 cleaves the COOH-terminal residue of the decapeptide. Ang I thus generates Ang (1-9) which is subsequently converted into Ang (1-7) [36, 37]. It shows a greater affinity for Ang II in comparison with Ang I. ACE2 has an important role in counterregulating the actions of the well-documented ACE/Ang II/AT1R axis which has a vasoconstrictive effect. ACE2 cleaves Ang II to Ang (1–7) which promotes vasodilation and has beneficial effects on cardiovascular diseases [38, 39]. Injection of Ang (1–7) into the brain resulted in a fall in arterial blood pressure indicating the central role of Ang (1–7) to counteract the rise in BP by Ang II. Thus, imbalance between ACE and ACE2 would affect blood pressure [40]. Local inhibition of brain ACE2 induces a reduction in baroreflex sensitivity. ACE2 is also the functional receptor for coronavirus associated with the acute respiratory syndrome, i.e., SARS-CoV [38].

### 2.5. Other Converting Enzymes: Aminopeptidase

As per the literature, renin is present in a very minute concentration in the brain and alone cannot contribute to Ang II generation. Hence, alternative converting enzymes such as tonin, cathepsin G, and chymase are involved which bypass the Ang I step and directly convert AGT to Ang II. Cathepsin G is a serine protease located in the neutrophil leukocytes, red blood cell membranes, and intracellular granules. Renin and cathepsins have a similar structure, which in the absence of renin leads to the generation of Ang II. Tonin is a serine proteinase also called rat kallikrein 2 is present in the astrocytes and thalamus of the brain. In case of absence or limited release of renin in the brain, tonin is responsible for Ang II generation [41]. Another enzyme chymase, which is a serine protease and is released from the mast cell, is also involved in the generation of Ang II. The one derived from humans has the highest Ang II generation capacity as compared to rat and rabbit chymase [42]. Other cleaving enzymes are aminopeptidases that play a very important role in the regulation of the RAS, and their inhibition shows significant effects (discussed earlier). APA is also known as glutamyl aminopeptidase and hydrolyzes the N-terminal of acidic amino acids. APA converts Ang II to Ang III. APN is alanyl aminopeptidase which hydrolyzes neutral amino acid residues. APB also called arginine aminopeptidase hydrolyzes N-terminal basic amino acids [43].

### 2.6. Angiotensin Receptors

#### 2.6.1. AT1 Receptors

In brain RAS, four types of angiotensin receptors exist—AT1, AT2, AT4, and MasR. AT1 and AT2 receptors are G protein-coupled receptor kinase having 7 transmembrane subunits and are of prime importance. AT2 is 34% identical at the amino acid level with the AT1 receptor [44].

**Figure 1:**
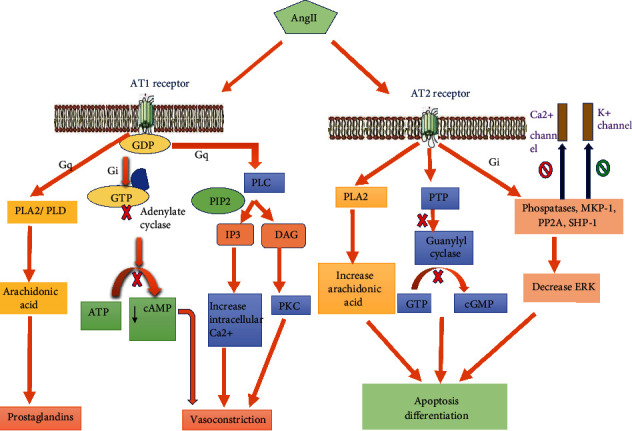
Signal transduction mechanism for angiotensin receptors—AT1 and AT2. Ang II binds to the AT1 receptor and shows vasoconstrictor action through Gi- and Gq-coupled mechanisms. Gi protein-coupled mechanism involves inhibition of adenylyl cyclase and phospholipase C stimulation coupled with Gq protein which activates secondary messengers like IP3 and diacylglycerol. Ca^2+^ released from the above pathway causes vasoconstriction. Vasoconstriction is also caused by protein kinase C from diacylglycerol and cAMP (vasodilator in nature) from the adenylyl cyclase inhibitory pathway. Gq-coupled mechanism activates phospholipase A2 and D, causing generation of arachidonic acid. Ang II binds with the AT2 receptor and by negative coupling with guanylyl cyclase shows Ca^2+^ inhibition and activation of the K^+^ channel. Also, the AT2 receptor acts through phospholipase A2 and stimulates arachidonic acid release. AT2 receptor stimulation activates several phosphatases like protein tyrosine phosphatase, MAP kinase phosphatase 1 (MKP-1), SH2-domain-containing phosphatase 1 (SHP-1), and serine threonine phosphatase 2A. When this phosphatase gets activated, there is inactivation of extracellular signal-regulated kinase (ERK), which leads to potassium channel opening and inhibition of T-type Ca^2+^ channels. Abbreviations: PLA: phospholipase A; PLC: phospholipase C; PLD: phospholipase D; IP3: inositol (1;4;5) triphosphate; DAG: diacylglycerol; PKC: protein kinase C; PTP: protein tyrosine phosphatase; PP2A: serine/threonine phosphatase 2A; ERK: extracellular signal-regulated kinase.

AT1 receptors are located in the organum vasculosum of the laminae terminalis (OVLT), the suprachiasmatic nucleus (SCN), the preoptic periventricular nucleus (PVN), and the median eminence. It consists of 359 amino acid sequences and has a molecular weight of 40-42 kDa. AT1 has 2 subtypes AT1A and AT1B which have a similar amino acid sequence but different pharmacological properties [45]. AT1A shows typical binding characteristics of the AT1 receptor, and AT1B has a 10000-fold higher affinity for the AT2 receptor antagonist PD123319. Ang II binds to the AT1 receptor by a transmembrane domain with the help of an extracellular loop. Ang II acts through AT1 receptor-Gi- and Gq-coupled mechanisms and causes vasoconstriction, hypertension, increased oxidative stress, and vasopressin release. A Gi protein-coupled mechanism involves inhibition of adenylyl cyclase and stimulation of phospholipase C (PLC). PLC forms Diacylglycerol (DAG) and cyclic AMP [46] (vasodilator in nature); it also releases protein kinase C (PKC) which results in vasoconstriction by the Gi inhibitory mechanism. Gq protein which activates the secondary messenger inositol triphosphate (IP3), and DAG releases phospholipase A2 (PLA2) and PLD, which in turn generate arachidonic acid having a role in inflammation (Figure 1). Icv infusion of the AT1 antagonist losartan inhibits hypertensive response to Ang II [47].

#### 2.6.2. AT2 Receptors

The AT2 receptor predominates in the midbrain mostly in the inferior olive, locus coeruleus, thalamic nuclei, medial amygdala, and molecular layers of the cerebellum. AT2 receptors are divided into two subtypes AT2A and AT2B. AT2A is reported to be present in the ventral thalamic nuclei, medial geniculate nuclei, and locus coeruleus and is sensitive to guanine nucleotides, pertussis toxin, and dithiothreitol. The AT2B subtype is located in the inferior olive and is found to be insensitive to the above-mentioned agents [48]. AT2 receptor activation causes lowering of BP and antihypertensive effect in neurogenic hypertension by the restoration of baroreflex [49].

**Figure 2:**
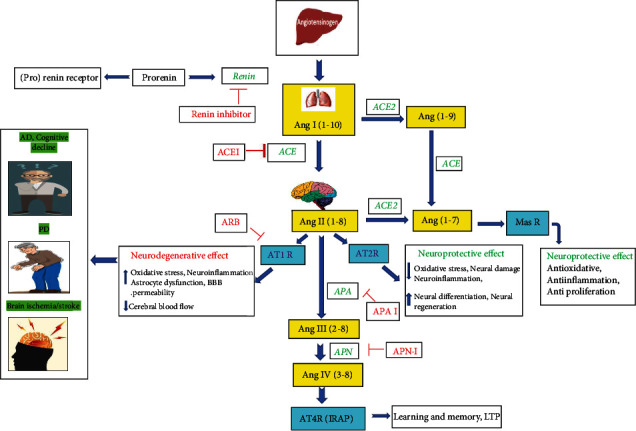
Function of angiotensin receptors in central nervous system-related disorders. Overview of brain RAS demonstrating the formation of its component in periphery and in the CNS. The diagram represents the specific converting enzymes involved for the conversion AGT to Ang IV, their receptors, and their role in various CNS disorders and protection. The RAS pathway initiates by conversion of angiotensinogen from the liver to Ang I in the lungs by sequential action of enzymes and prorenin-renin which also acts on a specific prorenin receptor. Ang I in the presence of ACE is further converted to Ang II, which gets fragmented into biological active form Ang III and IV which further acts on AT1, AT2, AT4, and MasR. The AT1 activation results in stress, neuroinflammation, and stroke. This effect is counteracted by the Ang II/AT2R and Ang (1-7)/MasR signaling pathway resulting in decrease in inflammatory cytokines and reduced ROS contributing to neuroprotection and cell proliferation. Specific inhibitors like ARBs, ACEI, and renin inhibitors have demonstrated to improve the pathological conditions. Abbreviations: Ang I: angiotensin I; Ang II: angiotensin II; Ang III: angiotensin III; Ang IV: angiotensin IV; ACE: angiotensin-converting enzyme; ACE 2: angiotensin-converting enzyme; APA: aminopeptidase A; APB: aminopeptidase B; APN: aminopeptidase N; MasR: Mas receptor; AT1: angiotensin receptor subtype 1; AT2: angiotensin receptor subtype 2; AT4: angiotensin receptor subtype 4; PD: Parkinson's disease; AD: Alzheimer's disease.

AT2 receptors are found in the fetus CNS and play an important role in the differentiation and development of CNS. They also contribute to repairing damaged DNA [50]. Ang II activates the AT2 receptor and causes K^+^ channel activation and inhibits Ca^2+^ release. It stimulates arachidonic acid release by acting through PLA2 and activates several phosphatases like protein tyrosine phosphatase, MAP kinase phosphatase 1, SH2-domain-containing phosphatase 1 (SHP-1), and serine-threonine phosphatase 2A [51–53]. This molecular-level action causes numerous effects like differentiation, vasodilation, and apoptosis. AT2 receptor activation therefore produces a neuroprotective effect (Figure 2)

#### 2.6.3. AT4 Receptors

AT4 receptors along with cholinergic neurons are present in the brain in the cerebral cortex and hippocampus, which are involved in cognition (memory formation), sensory, and motor performances [54, 55]. It increases cholinergic transmission and improves cognitive improvement abilities [56]. It is an insulin-regulated aminopeptidase (IRAP), and it shows its effect after binding to the ligand. Ang IV shows its pressor response through the AT1 receptor [57]. Ligands binding to AT1 and AT2 receptors do not bind to AT4 receptors proving that AT4 is not a GPCR but a member of the growth factor or cytokine family of receptors. The signal transduction mechanism of the AT4 receptor is still unclear for brain RAS [58].

#### 2.6.4. Mitochondrial Assembly Receptor (MasR)

MasR initially known as protooncogene is a G protein-coupled receptor. Mas expression is found in the heart, kidney, lung, liver, spleen, tongue, and skeletal muscle and excessively in the brain [59]. In the brain, they are specifically present in the NTS, RVLM, caudal ventrolateral medulla (CVLM), inferior olive, parvo- and magnocellular portions of the PVN, supraoptic nucleus, and lateral preoptic area [46]. The endogenous activation ligand for MasR is angiotensin (1-7). It is a negative regulator of Ang II-activated AT1R [35, 39]. Hence, the MasR agonist shows similar effects as Ang II receptor antagonists. The discovery of MasR led to the concept of two arms of RAS showing different actions: one comprising classic ACE/Ang II/AT1R (vasoconstrictive, hypertensive, proliferative, and fibrotic) and the other comprising ACE2/Ang (1-7)/Mas (provasodilatory, antihypertensive, antifibrotic, and antigrowth) [60]. The latter one plays an important role in the critical component of pulmonary systems, gastric mucosa, and cancer. Recently, experimental and clinical pieces of evidence suggested that Ang (1-7) or Mas analogs exhibit strong anti-inflammatory responses mediated by SARS-CoV-2 [61, 62]. The details have been discussed in the later part of the review.

### 2.7. Role of Brain RAS in the Regulation of Various Physiological Activities and the Drugs Acting on It

#### 2.7.1. Regulation of Cardiovascular Function through the Brain

Ang II acts as a neurotransmitter by activating its subtypes in different regions of the brain and thereby regulating neurogenic hypertension by sympathetic activation and baroreflexes. Brain RAS along with the sympathetic nervous system (SNS) and vasopressin release plays a crucial role in managing hypertension [63]. The release of vasopressin occurs from the hypothalamo-pituitary system while SNS activation occurs by the AT2 receptor via Ang II and Ang III. This leads to stimulation of pressor response and inhibition of baroreflex which causes an increase in the release of AVP into circulation and leads to increased BP. The central role of BP modulation by brain RAS is further confirmed by the fact that APA- and APN-specific inhibitors mainly acting through Ang III produce a fall in BP. Secondly, brain RAS acts by synaptic inhibition of baroreflex at the level of NTS and increased sympathetic nerve activity [25, 34]. NTS has an important role in the central feedback regulation of BP. At the moment of fluctuation of BP, aortic arch and carotid sinus baroreceptors get activated and send the signal to the NTS. It processes the signal and modulates synaptic output through initiating a relay from the caudal ventrolateral medulla and finally through RVLM. Thus, increased neuronal activity in RVLM or decreased baroreflex sensitivity at NTS can alter the sympathetic output. Inhibiting brain RAS by AT1 blockade in the brain leads to a decrease in blood pressure. However, there is need of preclinical studies where an angiotensin inhibitor like cardioprotector is required.

### 2.8. Role of Brain RAS in Thirst, Sodium Uptake, and Vasopressin Release

#### 2.8.1. Modulation of Thirst

Brain RAS influences BP by modulating synaptic nerve activity and stimulates behavioral changes that result in salt intake and initiation of thirst. Dehydration can be classified into extracellular (i.e., volumetric) or intracellular (i.e., osmotic). Brain structures involved in drinking behavior are the lamina terminalis and anteroventral third cerebral ventricle region (AV3V) including the SFO, OVLT, MnPO, and periventricular preoptic nuclei (PePO) which show high AT1 mRNA expression and high density of AT1 binding sites [25]. Volumetric dehydration caused by hemorrhage results in a decrease in blood volume and leads to baro/volume receptors in the kidney for renin release. It results in the start of a cascade of events that produce Ang II which plays an important role in the initiation of thirst [64]. When angiotensin reaches its threshold level (500 pg/ml by 48 h of dehydration), it penetrates the capillaries of SFO and OVLT. AT1 receptor expression causes activation of detectors in the arch of the aorta, carotid sinus, and great veins that send signals to the brain. It stimulates the thirst center in the brain to initiates a search for water, and antidiuretic hormone (ADH) is released. It results in decreased urine production and increased Na^+^ ingestion (sodium appetite). Extracellular dehydration thus increases thirst and sodium appetite.

#### 2.8.2. Sodium Uptake

Aldosterone is the hormone of sodium regulation, which plays an important role in the brain by sensitizing specific areas (hypothalamus and hindbrain) and affects circulating levels of Ang II. It also acts in the kidney via the distal tubule and collecting duct through stimulation of the sodium-potassium ATPase pump. The combined effect of aldosterone in the brain and kidney stimulates sodium appetite by inhibiting sodium excretion via the kidney and increasing thirst [64].

#### 2.8.3. Vasopressin Release

In the case of severe dehydration or hypovolemia, circulating angiotensin stimulates AT1A receptors in SFO and OVLT leading to activation of neuronal outputs from angiotensin neurons to PVN and SON, hence the release of angiotensin. This causes to increase the firing rate of vasopressin neurons and release of AVP from the posterior pituitary into circulation thereby combating fall in BP [65]. Vasopressin is also released when Ang II is injected in the brain and further activates the somatic nervous system and inhibits the baroreceptor reflex [66]. Angiotensin through vasopressin also promotes thermoregulation as observed by Ang II-facilitated heat loss in rats due to tail skin vasodilatation and by stimulation of central AVP release. Icv administration of the AT1 antagonist losartan in rats which are preexposed to a hot environment for 1 h showed the inhibition of the thermoregulatory cooling mechanism. It prevents splanchnic nerve activity and reduces the distribution of blood to the skin [65].

#### 2.8.4. Role of Brain RAS in Stroke (Neuroprotection)

Brain RAS has a much wider range of neural effects and is involved in Alzheimer's disease (AD), stroke, memory, learning, alcoholism, and stress [67]. Reduction or interruption of cerebral blood flow results in ischemic stroke. It is followed by neuronal necrosis and brain apoptosis resulting in serious damage to surrounding cells. Ang II stimulates the AT2 receptor and shows stroke-protective effects. Angiotensin receptor blockers (ARBs) show a neuroprotective effect by selectively blocking AT1 receptors leading to a decrease in local vasoconstriction. Further, free Ang II activates more AT2 receptors causing local vasodilation and decreases local brain ischemia, thus limiting volume and extent of brain loss. Preclinical studies using the middle cerebral artery occlusion (MCAO) model showed complete blockade of brain AT1 receptors, whereas when treated with ARBs, it exhibited neuroprotective effects in stroke, without having any effect on blood pressure [68]. Antihypertensive treatments, with different combinations of ACEI (perindopril), AT1 receptor antagonist, *β*-blocker, and diuretic treatments, are used clinically for controlling stroke [69]. ARBs acting on AT1R have also been shown to stimulate peroxisome proliferator-activated receptor gamma (PPAR*γ*). Therefore, blocking AT1R or/and activating PPAR*γ* may cause cerebral protection [70] (Figure 2). Telmisartan, ramipril, candesartan, and perindopril have been studied under clinical trials named PRoFESS, HOPE, HOPE-3, and PROGRESS, respectively, and found to be significantly effective. The details of the studies are mentioned in Table 1.

#### 2.8.5. Role of Brain RAS in Neuronal Damage

In brain RAS, Ang II plays an important role in neuronal damage, mainly acting through the dopaminergic neurons (nigrostriatal system) and microglial cells (inflammatory cells). It acts through the Ang II-mediated AT1/NOx axis and generates high-level superoxide ROS. Ang II acting through the AT1 receptor activates the NADPH-oxidase complex, which mediates various oxidative stress (OS) and inflammatory processes resulting in tissue damage leading to degenerative diseases [71, 72]. AT1-induced NOx activation is initiated by protein kinase C (PKC) in microglial cells [73]. Administration of ACEI and ARBs blocked the action of Ang II and showed a reduction in neurotoxin-induced levels of protein oxidation and lipid peroxidation and dopaminergic neuron protection [74] (Figure 2).

#### 2.8.6. Parkinson's Disease (PD)

PD is a neurodegenerative, movement disorder that occurs due to the progressive loss of dopaminergic neurons. Brain dopamine receptors are present in the substantia nigra pars compacta (SNPc) and striatum which has also shown the presence of the AT1 receptor [75]. Ang II activates NOx complex-stimulated superoxide generation by inflamed cells causing the death of dopaminergic neurons. ARBs (telmisartan, candesartan, and losartan) and ACEI (captopril, perindopril) are found to be potent against preclinical models of PD, although there are very limited clinical studies available [76–78].

#### 2.8.7. Role of Brain RAS in Memory Facilitation

Ang IV and cholinergic neurons are closely associated with the neocortex and hippocampus, which are involved in cognitive processing. Conversion of Ang II to Ang IV results in a memory-enhancing effect. The Ang IV analog Nle1-AngIV facilitates long-term potentiation (LTP) in learning and memory [79]. Elevated brain Ang II in AD interferes with Ach release from the cortex and affects cognitive functions. AT1R blockade by ARBs (telmisartan, losartan, valsartan, candesartan, and olmesartan) facilitates additional unbound Ang II available for conversion to Ang III and further to Ang IV and facilitates learning and memory. Also, the synthesis of Ang II is decreased by ACEI (perindopril, ramipril) which consequently increases Ach release and also the synthesis of Ang IV. Thus, coadministration of ACEI with AT1 antagonists prevents the formation and action of Ang II and enhances the formation of Ang IV, and it has been proven for its memory-enhancing property [80].

#### 2.8.8. Role of Brain RAS in AD and Dementia

AD is a multifactorial, complex, neurodegenerative disease that leads to dementia characterized by deposits of amyloid *β* (A*β*) (1-42), hyperphosphorylation of microtubule-associated protein tau, cholinergic neuronal loss, neuroinflammation, and mitochondrial damage [81]. Overexpression of ACE is observed in the hippocampus, frontal cortex, and caudate nucleus in patients with AD. There are contradictory effects observed for ACEIs in the treatment of Alzheimer's disease (AD) which are discussed below in detail. Some studies claim ACE inhibitors to be detrimental as ACE converts A*β*1–42 to A*β*1–40 (neuroprotective). Other studies have reported ACEI to be beneficial. It improved cerebral blood flow due to inhibition of the formation of Ang II. ACEI is also demonstrated to inhibit the release of inflammatory cytokines thereby attenuating neurodegeneration. ACE inhibition by Icv administration of perindopril has shown to ameliorate neurodegeneration [82, 83]. ARBs, namely, telmisartan, losartan, candesartan, valsartan, olmesartan, and eprosartan, by their inhibitory action on AT1R have shown to attenuate AD condition. They show their effect by the decrease in amyloidosis, microglial activation, reduced neuroinflammation, and improved cerebral blood flow. Candesartan and telmisartan have been studied clinically. A comparative clinical study of lisinopril with that of candesartan in 141 patients under the CALIBREX study demonstrated improved cognitive performance in the candesartan arm than lisinopril. With telmisartan's virtue of efficacy in preclinical studies under a clinical trial sponsored by the Alzheimer's Drug Discovery Foundation with 150 patients, the trial is open labelled and comparative with perindopril; it is expected to finish by 2022 (Table 1).

Kehoe et al. [84] studied human postmortem brain tissue and CSF of AD, diseased, and noncontrolled patients. The authors measured ACE1 and ACE2 activity by using an ACE1-specific fluorogenic peptide substrate (Abz-Frk(Dnp)-P) and ACE2-specific fluorogenic peptide substrate (mca-APK(Dnp) in the presence of a specific ACE1 inhibitor, captopril, and a ACE2 inhibitor, MLN4760, respectively. Angiotensin I and II and Ang (1-7) were measured by the direct ELISA method. The authors found ACE overactivity in brain RAS, and postmortem CSF in AD is mirrored in antemortem CSF. CSF-ACE1 in AD is higher in CSF-t-tau and CSF-p-tau but not CSF-A*β*-42. In contrast to brain tissues, ACE2 positively correlates with ACE1 activity in post- and antemortem CSF (inverse correlation in brain and brain tissue). Brain angiotensin changes in AD are not reflected in CSF. These findings indicated that some markers of brain RAS are mirrored in CSF; however, the relationship is complex. The recent research work by Evans et al. [85] has indicated beneficial effects of diminazene aceturate (ACE2 activator) by enhancement of brain ACE2 activity thereby lowering hippocampal A*β* and hence restoring cognition in symptomatic Tg2576 mice.

#### 2.8.9. Role of Brain RAS in Stress, HPA Axis Regulation, and Depression

AT1 receptors are majorly distributed in areas that control stress response such as SFO and the median eminence in the hypothalamic PVN, anterior pituitary gland, amygdala and septal nuclei, hippocampus, and NTS [86]. Stress results in the formation of Ang II in the thalamus and other parts of the brain that contribute to catecholamine release [87].

Brain RAS has already proven to have a role in the pathophysiology of depression [70]. As AT1 and AT2 receptors are found in the hypothalamus-pituitary-adrenal (HPA) [88] axis, HPA plays an important role in stress and stress-related behavior. Activation of corticotropin-releasing hormone (CRH) gene expression was found via AT1 receptor activation in immobilization-induced stress. Treatment with ARB (telmisartan, candesartan, and valsartan) or ACE inhibitors reduces CRH-induced adrenocorticotropic hormone (ACTH) and corticosterone release in spontaneously hypertensive rats. They also decreased pituitary sensitivity to CRH and reduced hypothalamic CRH expression which ultimately led to the reduction of stress [89] (Table 1). However, preclinical ARBs and ACEI are found to be effective. Clinical studies on them with respect to depression are still awaited.

#### 2.8.10. Role of Brain RAS in Alcohol Addiction

Alcohol consumption is majorly dependent on social aspects alongside genetic factors that play a crucial role in the pathogenesis of alcohol addiction. Experimental studies suggested that there is a direct correlation between endogenous Ang II level and voluntary alcohol consumption [90]. The reward circuitry (activated due to alcohol consumption) of the brain has dopamine as a critical component; accordingly, genetically modified D2 receptor-deficient mice have been shown to drink less alcohol [91]. Angiotensin receptors are expressed in the dopaminergic dominated nucleus accumbens; also, Ang II is demonstrated to stimulate dopamine release in the brain [92]. The central administration of Ang II to the experimental animal increased alcohol intake. ACE inhibitors, captopril and enalapril, therefore are found to prevent alcohol consumption [93].

#### 2.8.11. Role of Brain RAS in Pain

Pain is defined as hyperexcitation of sensory neurons that arises because of injury, trauma, inflammation, and nerve damage. Ang II injection into mouse hind paw has shown the development of peripheral pain. The amygdala, hypothalamus, and frontoparietal cortex are considered to be important parts in the relay of pain which also expresses RAS receptors. The drugs blocking RAS have been shown to inhibit and show potential benefits in central pain originating from either the brain or spinal cord such as migraine and neuropathy pain. The mechanism predicted is inhibition of cytokines and also due to the release of endogenous opioids. The literature also reports contradictory effects of brain RAS and ACEI which has shown an algesic effect due to inhibition of metabolism of bradykinin and substance P, while recent studies showed the antinociceptive effect of ACEI and ARBs by attenuating substance P, NO, and calcitonin gene-related peptide (CGRP). Newer drug candidates such as AT2R antagonists, EMA 200, EMA 300, EMA 401 have shown to inhibit P38 and p42/p44 MAPK; hence, they are found to be beneficial in neuropathic pain; however, the recent article published in June 2020 indicated that the AT2 receptor does not have any role to play in pain modulation [72, 94].

The early phase IIb clinical study on EMA 401, an AT2R antagonist, was found to be effective in neuropathic pain (Spinifex Pharmaceuticals) though the detailed clinical study results are awaited as the phase II clinical trial EPHENE was terminated due to lack of preclinical safety data (Table 1).

There is a lack of literature, and the unavailability of clinical trials on these molecules makes them obscure. Moreover, poor penetrability through BBB is another challenge to target brain AT2 receptors. Hence, there is a need to synthesize and develop more analogs and perform exhaustive preclinical studies to explore their potential in CNS-mediated pain-related disorders [95].

#### 2.8.12. SARS-CoV-2 Infection and Neurological Manifestations

Current threat of COVID-19, which has agitated the whole world, is an infectious disease caused by severe acute respiratory syndrome nCoV-2 (SARS CoV-2). COVID-19 has caused enormous deaths which have been reported worldwide. Symptoms associated resembles normal viral infection except in severe condition; it may develop to pneumonia, acute respiratory distress syndrome (ARDS), and multiorgan failure. SARS-CoV-2 consists of S protein (structural protein) which is multifunctional and plays a vital role in host receptor binding and pathogenesis of the virus. Postentry, there are a release of the viral RNA genome in the cytoplasm and translation into 2 polyproteins as well as structural proteins, and later replication of the viral genome begins. The membrane of the endoplasmic reticulum (ER) or Golgi is then inserted with newly formed unglycosylated proteins, and then, development of viral particles into the ER-Golgi intermediate compartment takes place followed by fusion of the plasma membrane with vesicles containing viral particles leading to the release of the virus. Apart from lung and cardiovascular tissues, newly assembled SARS-CoV-2 virions infect the ACE2-expressing cells on neuronal and glial cells in the brain. It also enters the brain predominantly through the olfactory mucosa (the first feature is the development of anosmia) and could spread through neuroanatomical interconnection. Within the CNS, the brain stem is worst affected leading to respiration and cardiovascular impairment and death. The acute infection of the brain is characterized by delirium followed by severe conditions such as unconsciousness, cerebrovascular events (stroke), encephalopathy, and seizures. The chronic effects of nCoV-2 infection include demyelization, dementia, and neuropsychological and neurodegenerative diseases [96]. Although the healthcare workers are now well versed with acute neurological events associated with nCoV-2, the post-COVID CNS complications must not be ignored that might occur as posttraumatic stress disorder that may invade the future well-being.

## 3. Conclusion

Since the conception of brain RAS in the year 1898, continuous research has been done in the field. It is now very well established that brain RAS plays a crucial role in CNS-controlled cardiovascular function, thirst, and maintenance of sodium level; also, it exhibits a prominent role in CNS itself. It is a neuroprotectant, observed via activation of the AT2 receptor; on the contrary, AT1 receptor activation has been shown to induce oxidative stress. Therefore, AT2 receptor agonists and AT1 receptor antagonists play a crucial role in cerebral protection. Similarly, blocking of AT1 receptors on dopaminergic neurons by ARBs and ACEI demonstrated a potent anti-PD effect. The role of Ang IV in the memory-enhancing effect is well established, though the invention of the Ang IV agonist is still awaited. ACE overexpression is found to be associated with AD, and its inhibition by perindopril and captopril has shown a promising effect in AD preclinically. The myriad of clinical trials on ARBs and ACEI are evident that indicate the possible clinical use of these drugs in AD in the near future. ACEI and ARBs are also found to be effective in depression by ameliorating the HPA axis. Recently, the role of brain RAS has been studied in the development of addiction. There is scope for more research on brain RAS in addiction and substance abuse, epilepsy, stress-related disorder, and psychosis. Recent literature also reported the analgesic effect of AT2R antagonists though still there is a need for preclinical and clinical studies to prove its clinical benefit. With a plethora of preclinical studies available for brain RAS, the unavailability of clinical trials on molecules modulating brain RAS obscures them. We have summarized the preclinical and clinical research work carried out using angiotensin enzyme and receptor inhibitors. However, further studies are needed to unfold the uses of these molecules in various pathological conditions associated with brain RAS.

## Figures and Tables

**Table 1:** Summary of preclinical as well as clinical research work carried using angiotensin enzyme and receptor inhibitors.

Sr. no	Drug	Disease	Disease model used	Results obtained	References
1	Telmisartan 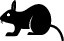	Alzheimer's Disease (AD) Neuroinflammation	Lipopolysaccharide- (LPS-) induced microglia 5XFAD mouse model	↓ amyloidosis (A*β* (1-40), amyloid plaques) ↑ cerebral blood flow ↓ neuroinflammation (↓A*β*O-IL-1*β*, TNF-*α*, and NF-*κ*b expression) ↓ microgliosis, ↓ astrogliosis	[97–99]
		Parkinson's disease (PD)	Rotenone-induced PD	↓ ER stress-mediated neuronal apoptosis	[100]
			MPTP-induced PD Catalepsy test	Neuroprotection against dopamine cell death (potent PPAR-*γ* activator) ↑ astroglial functions Restore dopaminergic function	[76, 101, 102]
		Cognitive impairment and vascular dementia	Chronic cerebral hypoperfusion, MWM	↑ spatial memory and learning ↓ neuronal loss ↑ PPARϒ	[103, 104]
		Acute and chronic stress	PAT, ORT, OFT, EPM, FST	HPA axis deactivation Upregulation of brain-derived neurotrophic factor (BDNF) gene expression	[105, 106]
		Diabetes-induced cognitive decline and depression	FST	Insulin sensitizer ↓ proinflammatory mediators Ameliorates the HPA axis function	[70, 107, 108]
		Cerebral ischemia-induced impairment of spatial memory	Eight-arm radial maze	Anti-inflammatory—partial PPAR*γ* agonist	[109]
		Traumatic brain injury (TBI)	Cortical impact injury in mice brain	Reduction in lesion volume and conservation of the hippocampus	[110]
		Epilepsy	Maximal electroshock and PTZ-induced seizure in mice	Antiepileptic	[88, 111, 112]

	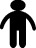	PRoFESS (Prevention Regimen for Effectively Avoiding Second Strokes) AD (ongoing) NCT02085265; NCT02471833	Patients—20332 (aged 67) (acute mild ischemic stroke and mildly elevated BP)	1360 patients appeared safe	[113, 114]

2	Losartan 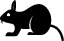	AD and cognitive impairment	A*β* peptide oligomerization assay, T maze, MWM, PAT, NOR, FST, EPM, MBT, Hebb-Williams maze (rectangular maze), amyloid-*β* precursor protein- (APP J20) induced AD	↓ neuroinflammation (↓ astrogliosis, ↓ microgliosis) ↓ plaque number and tau phosphorylation ↓ oxidative stress and inflammation ↑ cognition and memory, neurogenesis	[115–122]
		Parkinson's disease (PD)	MPTP- and 6-OHDA-induced PD in C57BL/6 mice (*α*-syn-based model)	Protect dopaminergic neurons against MPTP toxicity	[123]
		Learning and memory	OFT, EPM, SPT	Improve cognition function, ↓ oxidative stress and inflammation	[124, 125]
		Epilepsy Comorbid (hypertension and epilepsy) Cognitive impairment associated with epilepsy	Lithium pilocarpine-induced epilepsy Neuronal damage in the hippocampus Wire hang test Sticky paper test	Attenuate inflammation and oxidative stress, and exhibit neuroprotective effects Antiepileptic (neuroprotection selectively in the CA1 area of the hippocampus)	[126–128]
			Visual placing response Lithium pilocarpine-induced status epilepticus in rats	↓ microglial-mediated inflammation Attenuation of hippocampal neuronal loss	[129]
		Depression and anxiety	OFT EPM	Antidepressant and anxiolytic	[117, 130–132]
		TBI (traumatic brain injury)	Controlled cortical impact injury Rotarod test	↓ neuronal apoptosis and ER stress, enhance BBB integrity, PPAR-gamma activation ↑ cognitive/motor, ↓ cerebral blood flow	[133, 134]
		Neurocognitive alteration affecting long-term memory	Amphetamine-induced neurocognitive alteration in rats	Antipsychotic (↓ KYNA production)	[135]

	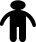	RADAR AD (ongoing) NCT02913664	Patients—228 (55 years with mild to moderate AD)	Assessment of rate of whole-brain atrophy, memory, cognitive function	[136]

3	Valsartan 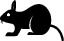	AD	Tg 2576 mouse model	↓ amyloidosis (↓ plaque load, A*β* oligomerization) Improves spatial learning	[98, 115, 137]
			APP/PS1 transgenic mouse model (intranasal)	↓ A*β*-inflammatory effects	[121]
		Cognitive impairment and amnesia	Icv STZ-induced cognitive impairment	Hippocampal neurogenesis Improves spatial learning ↓ acetylcholinesterase activity (increased acetylcholine)	[138]
		Depression and anxiety	CUMS, FST, OFT, NSF SPT	Antidepressant (↑ BDNF protein, ↓ noradrenaline and cortisone)	[139]

4	Candesartan 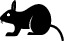	AD and cognitive impairment	LPS-induced microglia activation (5FXAD mouse model) Tg 2576 mouse model MWM	Anti-inflammatory, neuroprotective ↓ A*β* oligomerization ↓ neuroinflammation (↓ astrogliosis, ↓ microgliosis) Improved cognitive function (spatial memory, ↑ neurogenesis) Improved dementia	[99, 140, 141]
		PD	6-OHDA-induced PD in rats	Protection of dopaminergic neurons Inhibition of the synergetic action between Ang II and 6-OHDA	[142]
			Rotenone-induced PD in rats	Reduced neuronal loss and restored motor functioning.	[143]
			MPTP-induced PD in mice	PPAR-*γ* activation (partial agonism) Anti-inflammatory	[76]
		Cognitive impairment	Long-term repeated amphetamine administration	Neurorestorative effect ↑ expression of BDNF and TrkB	[135]
		Stress	Tight plastic tubes stress Inhibitory avoidance (IA) and object recognition (OR)	↓ stress ↑ memory	[144]
		TBI	Cortical impact injury in mice brain Hypertensive rat stroke model	↓ lesion volume and conservation of the hippocampus Upregulation of iNOS	[110, 145]
		Cognitive impairment postischemic stroke	NOR, SAP, PAT	Neurorestorative effect ↓ nonfatal stroke	[146–148]
		Depression and anxiety	LPS-induced brain injury in rats EPM	Anxiolytic and antidepressant	[131]

	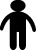	CALIBREX (NCT01984164)	Participants—176 (55 years or older) with mild cognitive impairment (MCI) and hypertension	Lower risk of dementia and AD	[149]
		SCOPE AD (ongoing) NCT02646982	Participants—4937 (70 to 89 years), evaluation of cognitive state by specific tests with mild-to-moderate hypertension	Hampered initiated cognitive decline	[150, 151]

5	Olmesartan 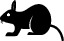	AD and cognition	MWM	Anti-inflammatory and antioxidative Prevent *β*-amyloid-induced vascular dysregulation (↓ ROS, MDA level) Improved A*β*-induced cognitive dysfunction	[98, 152]
		Cognitive impairment	PAT	↓ microvessel leakage (hippocampus and corpus callosum) Restored cognitive decline	[153]
		Epilepsy	MES- and PTZ-induced seizure in mice	Antiepileptic	[88]

6	Irbesartan 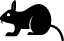	Schizophrenia	*In vitro* model using brain cortex slices	↓ KYNA production and KAT II activity in the rat brain cortex	[154]
		Depression	CUMS, FST, SPF	Antidepressant	[155]

7	Eprosartan 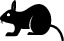	Early AD	C57bl/6j and A*β*PP/PS1/Alzheimer's disease mice Ang II infusion in the brain of AD mice	Improved cerebral blood flow and connectivity	[156]

	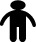	OSCAR	Patients—25745 (aged 50 yrs) with cognitive decline	Improvement of cognitive function	[157, 158]

8	Perindopril 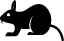	AD and cognitive impairment	Icv administration of amyloid-*β* (A*β*)_1-40_, induced AD mice Y-maze test	↓ oxidative stress Suppression of microglia/astrocyte activation	[82]
		PD	MPTP mouse model	Neuroprotection (inhibition of dopamine cell loss)	[159, 160]
		Vascular cognitive impairment	Chronic cerebral hypoperfusion in rats	Modulate BDNF Protect against neuroinflammation and oxidonitrosative stress	[161, 162]

	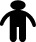	PROGRESS (Perindopril Protection against Recurrent Stroke Studies)	Participants—6105 (previous stroke or transient ischemic attack) Evaluation of cognitive state by specific tests	Improved cognition Reduced risk of AD	[163–166]
		CANTAB (Cambridge Neuropsychological Test Automated Battery)	Measurement of visuospatial, attention, and verbal memory, problem solving, learning, and reasoning	Improved cognitive function	[167, 168]

9	Captopril 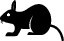	AD	LPS-induced microglia activation T-maze test	↓ amyloidogenic processing Antioxidant (↓ oxidative/nitrosative stress) ↓ inflammation, ↓ A*β* plaque accumulation	[169] [170]
		PD	MPTP-induced PD model	Protect dopamine cell from degeneration	[78, 171]
		Epilepsy	PTZ-induced seizures	↓ seizures and protection against postseizure neuronal injury in the hippocampus	[172]
		Depression	CUMS, chronic social defeat stress model	Antidepressant (BK-dependent activation of mTORC1)	[173]

10	Lisinopril 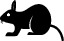	AD	STZ-induced dementia	PPAR-*γ* modulation	[174]
		Tardive dyskinesia	Haloperidol-induced stereotypic behavior	↓ oxidative damage and neuroinflammation	[175]

11	Trandolapril 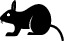	Huntington's disease	3-Nitropropionic acid-induced Huntington's disease EPM, MWM, narrow beam test, locomotor activity	Neuroprotection	[176]

12	Zofenopril 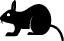	Cerebral ischemia	Bilateral coronary artery occlusion in rats. Biochemical analysis for the level of NO, SOD, and TAC	↓ TOS and MDA levels	[177]
		Epilepsy	Auditory stimulation model	Antiepileptic activity	[178]

13	Fosinopril 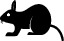	Amnesia	Scopolamine amnesia in rats, SPA, EPM, MWM	Antiamnesic activity	[179]
		Epilepsy	Audiogenic seizures	Antiepileptic	[178]

14	Ramipril 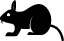 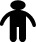	Cognitive impairment	Radiation-induced cognitive impairment, NOR	Potentiated ACE2/Ang-MasR axis ↓ microglial activation	[180]
		HOPE Stroke	Women with Vascular/Diabetic disorder—2480 (age—55 yrs and more) with vascular disease or diabetes	↓ incidence of fatal and nonfatal stroke	[181]

15	Aliskiren 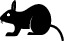	Ischemic stroke	Middle cerebral artery occlusion in mice	Neuroprotection, reduced infarct volume, and brain edema formation	[182] (Panahpour, 2019 #5)
		Epilepsy	MES-induced seizures in mice	Enhances the anticonvulsant effect of AEDs in mice	[183] (Panahpour, 2019 #5)

16	EMA 300, EMA 200 molecule 20 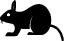	Pain	Unilateral chronic constriction injury of the sciatic nerve	Inhibit P38 and p42/p44 MAPK (inhibition of metabolism of bradykinin and substance P)	[184]
	EMA 401 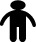	EMPHENE trial	Patients with postherpetic neuralgia	No beneficialeffects of AT2R are observed in pain	[185]

Abbreviations: AD: Alzheimer's disease; PD: Parkinson's disease; MPTP: 1-methyl-4-phenyl-1;2;3;6-tetrahydropyridine; PAT: passive avoidance test; ORT: object recognition test; OFT: open field test; EPM: elevated plus maze; FST: forced swim test; MES: maximal electroshock; MWM: Morris water maze; CUMS: chronic unpredictable model of stress; PTZ: pentylenetetrazol; NOR: novel object recognition; SPT: sucrose preference test; 6-OHDA: 6-hydroxyl dopamine.

## Data Availability

It is a review article; hence, there is no data which was generated in the laboratory.
